# Pembrolizumab-induced type-1 diabetes mellitus: clinical characteristics, therapeutic strategies, and prognosis from a retrospective analysis of 43 published cases

**DOI:** 10.3389/fendo.2026.1947327

**Published:** 2026-09-01

**Authors:** Jincai Guo, Yixiang Hu, Ya Liu, Yan Wang

**Affiliations:** 1 Department of Pharmacy, Changsha Stomatological Hospital, Changsha, China; 2 Department of Clinical Pharmacy, The Central Hospital of Xiangtan (The affiliated hospital of Hunan university), Xiangtan, China

**Keywords:** diabetic ketoacidosis, immune checkpoint inhibitor, immune-related adverse event, insulin dependence, pembrolizumab, type 1 diabetes mellitus

## Abstract

**Background:**

Pembrolizumab-induced type 1 diabetes mellitus (T1DM) is an uncommon but potentially life-threatening immune-related adverse event. Its clinical course is often abrupt, and evidence regarding its presentation, management, and long-term outcome remains largely derived from isolated case reports.

**Methods:**

Published case reports and case series describing pembrolizumab-induced T1DM were retrieved from PubMed, Embase, Web of Science, CNKI, and Wanfang Data from database inception to June 30, 2026, and retrospectively reviewed. Patient demographics, pembrolizumab exposure, clinical manifestations, laboratory findings, treatments, and outcomes were extracted and analyzed descriptively.

**Results:**

40 eligible articles identified 43 patients. The median age was 67 years (range, 12–87), and 24 patients (55.8%) were male. The median interval from pembrolizumab initiation to T1DM onset was 15 weeks (range, 3–104). Melanoma (34.9%) and lung cancer (23.3%) were the most common underlying malignancies, and 35 of 42 patients (83.3%) had no pre-existing diabetes. The predominant symptoms were fatigue or malaise (62.5%), polyuria, polydipsia, or thirst (57.5%), and nausea or vomiting (45.0%). Diabetic ketoacidosis occurred in 36 patients (83.7%). The median blood glucose level at presentation was 558 mg/dL (range, 277–1256), and the median HbA1c was 8.3% (range, 4.6–11.4). C-peptide was low in 30 of 34 patients (88.2%), whereas islet autoantibodies were detected in only 15 of 39 patients (38.5%). All patients required insulin therapy, and 27 (62.8%) received fluid or electrolyte replacement. Among 33 patients with available follow-up data, 32 (97.0%) had persistent insulin dependence or β-cell deficiency. Pembrolizumab was continued or restarted in 13 of 33 patients (39.4%); recurrent diabetic ketoacidosis was reported in one of these 13 patients.

**Conclusion:**

Pembrolizumab-induced T1DM is characterized by rapid and usually irreversible β-cell failure, frequent diabetic ketoacidosis, and persistent insulin dependence. Negative islet autoantibodies do not exclude the diagnosis. Glucose monitoring and prompt assessment of hyperglycemic symptoms are essential during and after treatment.

## Introduction

Immune checkpoint inhibitors have transformed the treatment of advanced malignancies by restoring antitumor T-cell activity. Programmed cell death protein 1 (PD-1) is an inhibitory receptor that restrains immune activation after binding to its ligands, PD-L1 and PD-L2. Pembrolizumab, a humanized IgG4 monoclonal antibody against PD-1, enhances antitumor immunity by interrupting this inhibitory pathway (1). Its clinical benefit has been established in melanoma, non-small-cell lung cancer, and several other malignancies (2, 3). However, disruption of immune tolerance may also cause immune-related adverse events (irAEs) involving multiple organs. Endocrine irAEs most commonly affect the thyroid and pituitary glands, whereas insulin-deficient diabetes is substantially less frequent (4).

Pembrolizumab-induced type 1 diabetes mellitus (T1DM) is uncommon but clinically important because of its abrupt onset and potential for life-threatening metabolic decompensation. Immune checkpoint inhibitor-associated diabetes is uncommon, with large cohort studies reporting an incidence of approximately 0.4–0.5% among ICI-treated patients, although estimates vary according to study population, treatment regimen, and diagnostic criteria (5–7). Unlike conventional autoimmune T1DM, which usually develops after a prolonged preclinical phase, checkpoint inhibitor-associated diabetes is characterized by rapid loss of pancreatic β-cell function (8). Patients frequently present with marked hyperglycemia, polyuria, polydipsia, fatigue, weight loss, nausea, or vomiting, and ketoacidosis (DKA) is the initial presentation in approximately 67%–71% of reported cases (9–11). Because hyperglycemia may evolve over a short period, glycated hemoglobin can be only moderately elevated despite severe hyperglycemia (12). C-peptide concentrations are usually low or undetectable at diagnosis or decline rapidly during follow-up, indicating profound endogenous insulin deficiency (13). The immunological profile of pembrolizumab-induced T1DM is heterogeneous. Islet autoantibodies, including antibodies against glutamic acid decarboxylase, insulinoma-associated antigen 2, insulin, islet cells, and zinc transporter 8, are detected in only a subset of patients; therefore, negative autoantibody findings do not exclude the diagnosis (10, 14, 15). Susceptible human leukocyte antigen genotypes and pre-existing islet autoimmunity may contribute to earlier disease development in some patients, although neither is consistently observed. The interval between pembrolizumab initiation and T1DM onset is also highly variable (16). Although many cases occur within the first several months of therapy, diabetes may develop after prolonged exposure or even months after pembrolizumab discontinuation (17).

The underlying mechanism remains incompletely understood. The PD-1/PD-L1 pathway contributes to peripheral immune tolerance in pancreatic islets. Experimental evidence indicates that interferon-γ-induced PD-L1 expression on β cells can restrain autoreactive T-cell activity, whereas PD-1 blockade may remove this protective signal and promote immune-mediated β-cell destruction (18, 19). Once severe insulin deficiency develops, recovery of pancreatic function is uncommon. Acute DKA requires intravenous insulin, fluid resuscitation, and electrolyte correction, followed by long-term subcutaneous insulin replacement (20). Corticosteroids, although widely used for other irAEs, have not been shown to restore β-cell function in established checkpoint inhibitor-associated T1DM (21). Most affected patients therefore remain permanently insulin dependent.

Current evidence on pembrolizumab-induced T1DM is derived mainly from individual case reports and small case series. Previous reviews have frequently pooled patients exposed to different PD-1, PD-L1, and CTLA-4 inhibitors, making pembrolizumab-specific characteristics difficult to define. Important uncertainties remain regarding its clinical presentation, biochemical phenotype, management, reversibility, and the safety of continuing or restarting pembrolizumab. We therefore conducted a literature-based retrospective analysis of 43 cases to characterize the clinical features, therapeutic strategies, and prognosis of pembrolizumab-induced T1DM, with the aim of facilitating earlier recognition and supporting individualized management.

## Methods

### Study design and search strategy

This study was a literature-based retrospective analysis of published cases of pembrolizumab-induced T1DM. A systematic search of PubMed, Embase, Web of Science, China National Knowledge Infrastructure (CNKI), and Wanfang Data was conducted from database inception to June 30, 2026. Search terms included combinations of “pembrolizumab,” “Keytruda,” “immune checkpoint inhibitor,” “type 1 diabetes mellitus,” “autoimmune diabetes,” “insulin-dependent diabetes,” “fulminant diabetes,” “diabetic ketoacidosis,” and “hyperglycemia,” together with their corresponding Chinese terms. No language restrictions were applied, and no human-study or publication-type filters were used at the database-search stage. Chinese databases were searched using corresponding Chinese terms. Case reports, case series, and individually identifiable cases reported within clinical studies were considered eligible.

### Eligibility criteria

Reports were included when they met the following criteria (1): pembrolizumab was administered before the onset of diabetes (2); the patient was diagnosed by the original authors with pembrolizumab-associated T1DM, immune checkpoint inhibitor-associated diabetes, acute-onset T1DM, or fulminant T1DM (3); individual-level clinical information was available; and (4) the temporal relationship between pembrolizumab exposure and diabetes onset could be assessed. Patients with pre-existing type 2 diabetes mellitus or prediabetes were retained when the report documented an abrupt deterioration in glycemic control accompanied by newly developed insulin dependence, substantial loss of C-peptide secretion, or other evidence supporting pembrolizumab-associated β-cell failure. Cases occurring after pembrolizumab discontinuation were also eligible when the temporal relationship remained clinically plausible. Reviews, mechanistic or animal studies, duplicate publications, reports involving checkpoint inhibitors other than pembrolizumab, conference abstracts lacking sufficient individual-level clinical information, and cases with insufficient individual data were excluded. Patients with established T1DM before pembrolizumab treatment or with worsening pre-existing diabetes without convincing evidence of newly developed insulin deficiency were also excluded.

### Study selection and data extraction

Case reports, case series, and individually identifiable cases from clinical studies were eligible when pembrolizumab exposure preceded the onset of T1DM and sufficient patient-level clinical information was available for assessment. Patients with pre-existing type 2 diabetes mellitus or prediabetes were included only when the report documented an abrupt deterioration in glycemic control accompanied by newly developed insulin dependence, markedly reduced C-peptide secretion, or other evidence of acute β-cell failure. Cases occurring after pembrolizumab discontinuation were also retained when the temporal relationship remained clinically plausible. Reviews, mechanistic or animal studies, duplicate publications, reports involving immune checkpoint inhibitors other than pembrolizumab, cases with established T1DM before treatment, and reports lacking adequate individual data were excluded. A standardized data-extraction form was used to collect age, sex, country, underlying malignancy, medical history, pre-existing glucose metabolism status, pembrolizumab dose and treatment duration, concomitant anticancer therapy, time to diabetes onset, presenting symptoms, DKA status, and laboratory findings. Data on blood glucose, HbA1c, C-peptide, ketones, blood pH, bicarbonate, anion gap, and islet autoantibodies were recorded when available. Therapeutic interventions, pembrolizumab discontinuation or continuation, tumor response, diabetes recovery, insulin dependence, rechallenge outcomes, and causality assessment were also extracted. Missing information was recorded as not reported and was not imputed.

### Quality assessment of case reports

The quality of the included case reports and case series was independently evaluated using the Joanna Briggs Institute (JBI) Critical Appraisal Checklist for Case Reports, which consists of eight assessment domains (https://jbi.global/critical-appraisal-tools). Two reviewers independently assessed each item and categorized responses as “Yes,” “No,” “Unclear,” or “Not applicable.” Discrepancies between reviewers were resolved through discussion and consensus, with a third reviewer consulted when necessary.

### Causality assessment

The causal association between pembrolizumab and T1DM was assessed using the World Health Organization–Uppsala Monitoring Centre system. Cases were classified as probable when there was a reasonable temporal relationship, alternative explanations were unlikely, and the clinical course was compatible with a drug-related event. Cases were classified as possible when the temporal relationship was reasonable but competing causes could not be fully excluded or the effect of pembrolizumab withdrawal was uncertain. Any disagreements between the two investigators were resolved through discussion and consensus, with a third investigator consulted when necessary.

### Statistical analysis

Statistical analyses were performed using SPSS version 23.0. Continuous variables were described using medians and ranges, and categorical variables were presented as absolute numbers with corresponding percentages.

## Results

### Study selection

**Figure 1:**
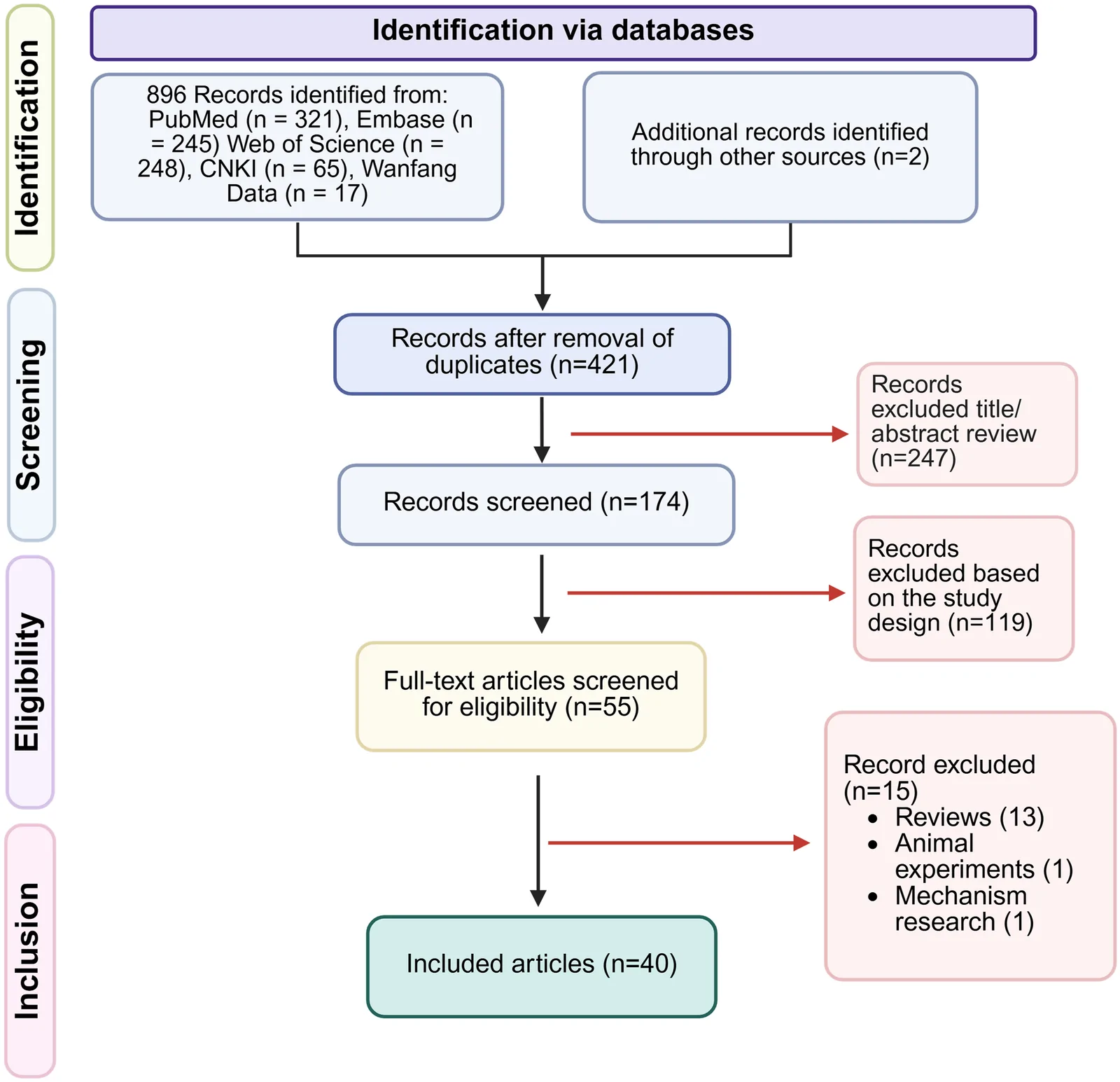
Flowchart illustrating the study selection process for inclusion.

896 records were identified through database searching and an additional 2 records were obtained from other sources (as shown in **Figure 1**). After duplicate removal and title/abstract screening, potentially eligible reports were assessed in full text. Reviews, animal or mechanistic studies, duplicate publications, non-pembrolizumab cases, and reports with insufficient patient-level data were excluded. Ultimately, 40 articles describing 43 patients with pembrolizumab-induced T1DM were included in the analysis (**Figure 1**), and their individual characteristics are presented in **Supplementary Table 1** (8–10, 12, 14, 22–56). Overall, most reports met the JBI criteria for acceptable quality, with adequate descriptions of patient characteristics, clinical presentation, diagnostic evaluation, treatment, outcomes, and key clinical lessons. Detailed item-level assessments are provided in **Supplementary Table 2**.

### Basic characteristics

As shown in **Table 1**, a total of 43 patients with pembrolizumab-induced T1DM were included in the analysis. Age was reported for 41 patients, with a median age of 67 years (range, 12–87 years). Twenty-four patients (55.8%) were male and 19 (44.2%) were female. The largest number of cases originated from the United States (15, 34.9%), followed by Japan (7, 16.3%), China (4, 9.3%), and South Korea (3, 7.0%). Two cases each were reported from Australia, Belgium, New Zealand, Portugal, and the United Kingdom, while France, Israel, Norway, and Spain contributed one case each. The median interval from pembrolizumab initiation to T1DM onset was 15 weeks (range, 3–104 weeks). Nineteen patients (44.2%) developed diabetes within 10 weeks, six (14.0%) between weeks 11 and 20, five (11.6%) between weeks 21 and 30, and 13 (30.2%) after more than 30 weeks. Melanoma was the most common indication for pembrolizumab (15, 34.9%), followed by lung cancer (10, 23.3%) and urothelial or bladder cancer (3, 7.0%). Among 42 patients with available baseline glucose status, 35 (83.3%) had no diabetes, two (4.8%) had prediabetes, and five (11.9%) had type 2 diabetes mellitus. Ten patients (23.3%) received concomitant anticancer agents. Pembrolizumab dosage was reported in 20 patients. Ten (50.0%) received 2 mg/kg, five (25.0%) received 200 mg every 3 weeks, four (20.0%) received 200 mg with an unspecified dosing interval, and one received another regimen.

**Table 1:** Basic characteristics of 43 patients with pembrolizumab−induced type−1 diabetes mellitus.

Parameter	Classification	Value
Gender (43)^a^	Male	24 (55.8%)
	Female	19 (44.2%)
Age (41)^a^	Years	67 (12, 87)^b^
Country (43)^a^	USA	15 (34.9%)
	Japan	7 (16.3%)
	China	4 (9.3%)
	South Korea	3 (7.0%)
	Australia	2 (4.7%)
	Belgium	2 (4.7%)
	New Zealand	2 (4.7%)
	Portugal	2 (4.7%)
	United Kingdom	2 (4.7%)
	France, Israel, Norway and Spain (each n = 1)	4 (9.3%)
Symptom onset time (43)^a^	Weeks	15 (3, 104)^b^
	1–10	19 (44.2%)
	11–20	6 (14.0%)
	21–30	5 (11.6%)
	>30	13 (30.2%)
Indication (43)^a^	Melanoma	15 (34.9%)
	Lung cancer	10 (23.3%)
	Urothelial/bladder cancer	3 (7.0%)
	Breast cancer	2 (4.7%)
	Esophageal/gastroesophageal junction cancer	2 (4.7%)
	Biliary tract cancer	2 (4.7%)
	Head and neck cancer	2 (4.7%)
	Hodgkin lymphoma, cardiac angiosarcoma, cervical cancer, renal carcinoma, pancreatic cancer, gastric cancer and Merkel cell carcinoma (each n = 1)	7 (16.3%)
Pre-existing glucose metabolism status (42)^a^	No diabetes	35 (83.3%)
	Prediabetes	2 (4.8%)
	Type 2 diabetes mellitus	5 (11.9%)
Pembrolizumab dosage (20)^a^	Weight-based dosing (2 mg/kg)	10 (50.0%)
	200 mg every 3 weeks	5 (25.0%)
	200 mg (frequency not reported)	4 (20.0%)
	Other dosage	1 (5.0%)
Concomitant anticancer medications (10)^a^	Tegafur, carboplatin, paclitaxel/nab-paclitaxel, pemetrexed, folinic acid, fluorouracil, oxaliplatin and lenvatinib	10 (23.3%)

^a^ Represents the number of patients with this parameter out of 43 patients.

^b^ Median (minimum, maximum).

### Clinical manifestations

As shown in **Table 2**, clinical symptoms were described in 40 patients. Fatigue, weakness, or malaise was the most frequently reported manifestation, occurring in 25 patients (62.5%). Polyuria, polydipsia, or increased thirst was observed in 23 patients (57.5%), and nausea or vomiting occurred in 18 (45.0%). Weight loss and dry mouth or dehydration were each reported in 10 patients (25.0%). Neurological or hemodynamic manifestations were less common but clinically important. Altered consciousness or confusion occurred in seven patients (17.5%), while dizziness, lightheadedness, or syncope was reported in five (12.5%). Respiratory signs, including tachypnea or Kussmaul respiration, were present in seven patients (17.5%). Abdominal pain and diarrhea each occurred in six patients (15.0%), and anorexia was reported in four (10.0%). Other less frequent manifestations included rash, headache, visual changes, palpitations, gastrointestinal bleeding, hypotension, and cold extremities. Diabetic ketoacidosis (DKA) was the predominant acute presentation. Thirty-six of the 43 patients (83.7%) presented with DKA, whereas seven patients (16.3%) developed pembrolizumab-associated T1DM without documented DKA. Among the seven patients without documented DKA, the median presenting glucose level was 500 mg/dL, and the median HbA1c was 7.1% among the six patients with available HbA1c data. These findings indicate that, in most reported cases, the diagnosis was established only after substantial metabolic decompensation had already occurred.

**Table 2:** Clinical manifestations and laboratory findings of 43 patients with pembrolizumab-induced type 1 diabetes mellitus.

Parameter	Classification	Value
Clinical symptoms (40)^a^	Fatigue/weakness/malaise	25 (62.5%)
	Polyuria/polydipsia/thirst	23 (57.5%)
	Nausea/vomiting	18 (45.0%)
	Weight loss	10 (25.0%)
	Dry mouth/dehydration	10 (25.0%)
	Altered consciousness/confusion	7 (17.5%)
	Respiratory signs (tachypnea/Kussmaul respiration)	7 (17.5%)
	Abdominal pain	6 (15.0%)
	Diarrhea	6 (15.0%)
	Dizziness/lightheadedness/syncope	5 (12.5%)
	Anorexia	4 (10.0%)
	Other symptoms: rash, headache, visual changes, palpitations, gastrointestinal bleeding, hypotension/cold extremities	9 (22.5%)
Diabetic ketoacidosis (43)^a^	Yes	36 (83.7%)
	No	7 (16.3%)
Laboratory tests		
Blood glucose (41)^a^	mg/dL	558 (277, 1256)^bc^
HbA1c (38)^a^	%	8.3 (4.6, 11.4)^bc^
C-peptide status (34)^a^	Low	30 (88.2%)
	Preserved at onset	4 (11.8%)
Ketone status (33)^a^	Positive/elevated	33 (100.0%)
Blood pH (28)^a^		7.17 (6.84, 7.43)^b^
Bicarbonate (30)^a^	mmol/L	9.0 (3.0, 22.0)^b^
Anion gap (19)^a^	mmol/L	26 (14, 39)^b^
Islet autoantibodies (39)^a^	Positive	15 (38.5%)
	Negative	24 (61.5%)
Anti-GAD antibody (39)^a^	Positive	12 (30.8%)
Anti-IA2 antibody (20)^a^	Positive	3 (15.0%)
ICA (14)^a^	Positive	2 (14.3%)
IAA/insulin autoantibody (16)^a^	Positive	1 (6.3%)

^a^ Represents the number of patients with this parameter out of 43 patients.

^b^ Median (minimum, maximum); ^c^Glucose values reported in mmol/L were converted to mg/dL, and HbA1c values reported in mmol/mol were converted to percentage units.

DKA, diabetic ketoacidosis; HbA1c, glycated hemoglobin; GAD, glutamic acid decarboxylase; IA-2, insulinoma-associated antigen 2; ICA, islet cell antibody; IAA, insulin autoantibody.

### Laboratory findings

Blood glucose values were available for 41 patients (**Table 2**). The median glucose concentration at presentation was 558 mg/dL, with a wide range from 277 to 1256 mg/dL. HbA1c was reported for 38 patients, with a median value of 8.3% (range, 4.6%–11.4%). The combination of severe hyperglycemia and, in some cases, only moderately elevated HbA1c was consistent with the relatively abrupt development of glucose dysregulation. C-peptide status was documented in 34 patients. Thirty patients (88.2%) had low or undetectable C-peptide concentrations, whereas four patients (11.8%) retained measurable secretion at presentation. In several of the latter cases, insulin secretion subsequently declined during follow-up. Ketone measurements were available for 33 patients, all of whom had positive or elevated ketone levels. Acid–base abnormalities were also prominent. Among the 28 patients with reported blood pH, the median value was 7.17 (range, 6.84–7.43). The median bicarbonate concentration was 9.0 mmol/L (range, 3.0–22.0 mmol/L; n=30), and the median anion gap was 26 mmol/L (range, 14–39 mmol/L; n=19). These findings reflected the high frequency and severity of ketoacidosis in the included cases. Islet autoantibody results were reported in 39 patients. At least one islet autoantibody was detected in 15 patients (38.5%), whereas 24 (61.5%) were autoantibody-negative. Anti-glutamic acid decarboxylase antibodies were the most frequently detected, occurring in 12 of 39 tested patients (30.8%). Anti-insulinoma-associated antigen 2 antibodies were positive in 3 of 20 patients (15.0%), islet cell antibodies in 2 of 14 (14.3%), and insulin autoantibodies in 1 of 16 (6.3%). Thus, most patients did not have detectable conventional islet autoantibodies despite clear evidence of insulin deficiency.

### Treatment and pembrolizumab management

All 43 patients received insulin therapy (**Table 3**). Fluid or electrolyte replacement was reported in 27 patients (62.8%). Six patients (14.0%) required thyroid hormone replacement, 5 (11.6%) received oral glucose-lowering agents, and 2 (4.7%) received adrenal hormone replacement. Corticosteroids were administered for other immune-related adverse events in 4 patients (9.3%). Three patients (7.0%) received corticosteroids as an attempted treatment for diabetes, but restoration of β-cell function was not documented. Information on subsequent pembrolizumab management was available for 33 patients. Pembrolizumab was continued or restarted in 13 patients (39.4%), whereas it was discontinued or not resumed in 20 (60.6%). Details regarding the timing of resumption, subsequent treatment cycles, and concurrent anticancer therapies were inconsistently reported in the original reports and could not be reliably summarized.

**Table 3:** Therapeutic strategies and clinical outcomes in 43 patients with pembrolizumab-induced type 1 diabetes mellitus.

Parameter	Classification	Value
Treatment (43)^a^	Insulin therapy	43 (100.0%)
	Fluid/electrolyte replacement	27 (62.8%)
	Thyroid hormone replacement	6 (14.0%)
	Oral glucose-lowering therapy	5 (11.6%)
	Corticosteroids for other irAEs	4 (9.3%)
	Corticosteroid trial for diabetes	3 (7.0%)
	Adrenal hormone replacement	2 (4.7%)
Pembrolizumab management (33)^a^	Continued/restarted	13 (39.4%)
	Discontinued/not resumed	20 (60.6%)
Outcome (43)^a^	Survived/clinically stabilized	38 (88.4%)
	Death (tumor related)	4 (9.3%)
	Transition to hospice	1 (2.3%)
Tumor response (19)^a^	Stable disease/response	15 (78.9%)
	Progression/no response	4 (21.1%)
Diabetes recovery (33)^a^	Insulin independence/recovery	1 (3.0%)
	Persistent insulin dependence/β-cell deficiency	32 (97.0%)
Rechallenge/continuation outcome (13)^a^	No recurrent DKA reported	12 (92.3%)
	Recurrent DKA	1 (7.7%)
WHO-UMC causality category (43)^b^	Probable	31 (72.1%)
	Possible	12 (27.9%)

^a^ Represents the number of cases describing this parameter out of 43 patients. Treatment categories were not mutually exclusive.

^b^ Under the World Health Organization–Uppsala Monitoring Centre (WHO–UMC) system, a “probable” case has a reasonable temporal relationship, is unlikely to be explained by alternative causes, and has a clinically reasonable response to withdrawal when applicable. A “possible” case has a reasonable temporal relationship, but alternative causes cannot be excluded or the response to withdrawal is unclear.

### Clinical outcomes

As shown in **Table 3**, overall clinical outcome was available for all 43 patients. Thirty-eight patients (88.4%) survived or achieved clinical stabilization following treatment. Four patients (9.3%) died, and one patient (2.3%) transitioned to hospice care. Death was not uniformly attributable to diabetes alone, as some patients had advanced malignancy or other severe complications. Tumor response was described in 19 patients. Fifteen patients (78.9%) had stable disease or an objective treatment response, while four (21.1%) had progressive disease or no response. These data were limited by incomplete oncological follow-up in the original reports. Long-term diabetes outcomes were available for 33 patients. Only one patient (3.0%) achieved insulin independence or recovery, whereas 32 patients (97.0%) remained insulin dependent or had persistent β-cell deficiency. The high proportion of persistent insulin dependence indicated that pembrolizumab-associated β-cell injury was usually irreversible once clinically apparent diabetes had developed. Among the 13 patients who continued or restarted pembrolizumab, no recurrent DKA was reported in 12 (92.3%). One patient (7.7%) developed recurrent DKA. However, these observations should be interpreted cautiously because of the small, selected sample and heterogeneous follow-up. According to the World Health Organization–Uppsala Monitoring Centre causality criteria, 31 cases (72.1%) were classified as probable and 12 (27.9%) as possible.

## Discussion

This pembrolizumab-specific analysis extends previous studies that pooled different immune checkpoint inhibitors and indicates that the clinical phenotype is not uniform (10). Our findings were broadly consistent with larger studies of ICI-associated diabetes. In a systematic analysis of 172 cases of ICI-associated diabetes, the median time to diabetes onset was 12 weeks, DKA occurred in 67.4%, and low C-peptide levels were observed in 91.8% of evaluable patients (11), findings broadly comparable with our analysis except for the higher frequency of DKA (83.7%). Overall, pembrolizumab-associated T1DM appears to share the major clinical features reported with other ICIs, although the wide onset interval, occasional preservation of C-peptide, and inconsistent autoantibody findings in our analysis suggest a spectrum ranging from fulminant β-cell failure to a more gradually evolving process (11, 15). Thus, not every patient fulfills conventional criteria for fulminant T1DM, even when DKA is the first manifestation. The relatively high frequency of DKA in our analysis may partly reflect publication bias toward severe cases rather than a pembrolizumab-specific feature. Fatigue, nausea, anorexia, and weight loss overlap with cancer-related symptoms, while polyuria and thirst may not be reported until metabolic deterioration is advanced (21). Marked hyperglycemia alongside a median HbA1c of 8.3% supports a relatively short period of glycemic deterioration in many patients. HbA1c alone is therefore insufficient to exclude evolving disease. Glucose should be checked throughout therapy, and ketones, bicarbonate, and blood gas analysis should be obtained promptly when hyperglycemia or compatible symptoms appear (57). Because delayed cases occurred after pembrolizumab withdrawal, vigilance should continue beyond the last infusion.

The laboratory pattern has important diagnostic implications. Nearly 90% of tested patients had low or undetectable C-peptide, confirming that insulin deficiency rather than isolated treatment-related insulin resistance was the dominant abnormality. However, four patients retained measurable secretion at onset. A preserved C-peptide value should therefore be interpreted against the simultaneous glucose concentration and repeated when suspicion remains high. Only 38.5% of patients had detectable islet autoantibodies. Autoantibody positivity may indicate pre-existing islet autoimmunity in some patients, but negative findings neither exclude the diagnosis nor provide a reliable universal screening strategy (16). Among the tested autoantibodies, anti-glutamic acid decarboxylase (GAD) antibodies were the most frequently detected, occurring in 30.8% of patients, consistent with previous ICI-associated diabetes reviews. Both autoantibody-positive and autoantibody-negative presentations have been documented in pembrolizumab-associated diabetes (27). PD-1 blockade may remove PD-L1-mediated protection of β cells, unleash autoreactive cytotoxic T cells, and accelerate β-cell destruction; variable autoantibodies and HLA backgrounds suggest heterogeneous immune pathways rather than a single classical autoimmune process (58). HLA susceptibility may also contribute to risk, with diabetes-associated haplotypes such as DR3-DQ2 and DR4-DQ8 reported in some ICI-DM cohorts, although their predictive value remains uncertain (59). The near-universal persistence of insulin dependence is clinically consequential. Only one of 33 patients with follow-up achieved insulin independence, suggesting that overt disease generally reflects advanced, irreversible β-cell loss. This also explains why corticosteroids differ from their role in many other irAEs. In the three patients treated with corticosteroids for diabetes, β-cell recovery was not documented; an earlier report likewise found persistently undetectable C-peptide despite high-dose prednisolone. Corticosteroids may additionally worsen hyperglycemia and increase insulin requirements. Management should therefore prioritize standard DKA treatment, structured insulin replacement, diabetes education, and prevention of recurrent metabolic decompensation rather than empirical immunosuppression (20, 58).

Whether pembrolizumab should be permanently discontinued after T1DM onset remains an important clinical consideration. Although 12 of 13 patients who continued or restarted pembrolizumab had no reported recurrent DKA, this finding should be interpreted cautiously because of the small sample size, substantial selection/publication bias, and variable follow-up duration. For context, previous studies of ICI rechallenge have reported recurrence of the same irAE in approximately 29%–32% of patients, although recurrence risk varies across organ systems (60, 61). Therefore, these data are insufficient to establish the safety of rechallenge, and treatment decisions should be individualized according to oncological benefit and metabolic stability. Tumor control was reported in 15 of 19 evaluable patients. Although endocrine irAEs have sometimes been associated with effective immune activation, these data cannot establish that diabetes predicts antitumor benefit because oncological outcomes were incomplete and selectively reported (62). Patients with favorable cancer responses may also have received longer pembrolizumab exposure and more intensive follow-up, increasing the opportunity to detect and report endocrine toxicity. This observation should therefore remain hypothesis-generating rather than being interpreted as a prognostic association.

### Limitations of the study

This study has several limitations. It relied on published case reports and case series, which are susceptible to publication and reporting bias and may overrepresent severe presentations. Clinical definitions, laboratory assays, follow-up duration, and reporting completeness varied across studies. Concomitant anticancer drugs, comorbidities, and other endocrine immune-related adverse events could not be fully excluded as contributors in some cases. The absence of a comparator group prevented estimation of incidence or identification of independent risk factors, while the small, highly selected rechallenge subgroup, variable follow-up duration, and incomplete reporting of rechallenge timing and subsequent treatment cycles limited reliable evaluation of rechallenge outcomes. Despite these limitations, the analysis delineates a consistent pattern of abrupt hyperglycemia, frequent DKA, marked insulin deficiency, and usually permanent insulin dependence. Earlier recognition and structured metabolic monitoring are therefore central to reducing preventable morbidity while preserving effective anticancer therapy whenever clinically appropriate.

## Conclusion

Our analysis provides clinically relevant insights into the recognition, management, and prognosis of pembrolizumab-induced T1DM. This adverse event should be considered when new-onset hyperglycemia or compatible symptoms develop during or after pembrolizumab treatment, particularly when accompanied by ketosis, metabolic acidosis, and reduced C-peptide secretion. Negative islet autoantibodies do not exclude the diagnosis. Although the optimal strategy for surveillance and early detection remains uncertain, baseline and periodic glucose/HbA1c monitoring, patient education regarding symptoms of hyperglycemia and DKA, and early ketone testing when hyperglycemia or compatible symptoms occur may facilitate earlier recognition. Prompt recognition and intervention, including fluid and electrolyte replacement for DKA and timely initiation of insulin therapy, are essential to reduce acute morbidity. Corticosteroids appear unlikely to restore β-cell function once overt insulin deficiency has developed, and most patients require long-term insulin replacement. Evidence regarding pembrolizumab continuation or rechallenge remains limited, and treatment decisions should therefore be individualized according to oncological benefit, glycemic control, coexisting immune-related adverse events, and the availability of close multidisciplinary follow-up.

## Data Availability

The original contributions presented in the study are included in the article/**Supplementary Material**. Further inquiries can be directed to the corresponding author.
